# Lack of Association between Methionine Synthase A2756G Polymorphism and Digestive System Cancer Risk: Evidence from 39327 Subjects

**DOI:** 10.1371/journal.pone.0061511

**Published:** 2013-04-16

**Authors:** Yuan Zhao, Zixian Chen, Yushui Ma, Qing Xia, Feng Zhang, Da Fu, Xiao-Feng Wang

**Affiliations:** 1 Department of Gastroenterology, Zhongshan Hospital, Fudan University, Shanghai, People’s Republic of China; 2 Department of Orthopaedic Surgery, Zhongshan Hospital, Fudan University, Shanghai, People’s Republic of China; 3 Key Laboratory of Stem Cell Biology, Institute of Health Sciences, Shanghai Institutes for Biological Sciences, Chinese Academy of Sciences, Shanghai, People’s Republic of China; Gentofte University Hospital, Denmark

## Abstract

**Background:**

Polymorphisms in genes involved in the metabolism of folate and methyl groups have been implicated with risk of digestive system cancer. Methionine synthase (MTR) plays a central role in folate metabolism, thereby affecting DNA methylation. The association between A2756G polymorphism (rs1805087) in MTR and digestive system cancer susceptibility was inconsistent in previous studies. To investigate this inconsistency, we performed this meta-analysis.

**Methods:**

Databases including Pubmed, EMBASE, ISI Web of Science and China National Knowledge Infrastructure (CNKI) were searched to find relevant studies. Odds ratios (ORs) with 95% confidence intervals (CIs) were used to assess the strength of association. Potential sources of heterogeneity were also assessed by subgroup analysis and meta-regression.

**Results:**

A total of 29 articles with 15,368 patients and 23,959 controls were included. We found no association between MTR A2756G polymorphism and digestive system cancer in overall population (G allele: OR = 1.03, 95% CI = 0.98–1.09, P = 0.25; dominant model: OR = 1.03, 95% CI = 0.97–1.10, P = 0.33; recessive model: OR = 1.02, 95% CI = 0.89–1.17, P = 0.79). In the stratified analyses according to cancer type, sample size and genotyping method, no evidence of any gene-disease association was obtained in almost all genetic models. However, marginal significant associations were found for East Asians and hospital-based studies.

**Conclusions:**

This meta-analysis suggests that there is no significant association between the MTR A2756G polymorphism and digestive system cancer risk.

## Introduction

It is predicted that by 2020, the number of new cases of cancer in the world will increase to more than 15 million, with deaths increasing to 12 million [1]. Digestive system cancers are the most common malignant tumors worldwide, with three million new cases each year (nearly 30% of all cancers) [1], [2]. The incidence of digestive system cancers will be constantly increasing, mainly due to trends in gastric cancer (GC) and colorectal cancer (CRC) [2]. In European countries, there were an estimated 0.91 million new cases of digestive system cancers (436,000 CRC and 149,000 GC) and 0.59 million deaths from these health care problems in 2008 [3]. In the majority of developing countries, the upward trends of mortality rates for digestive system cancers also have been observed [4], [5].

Methylation of the promoter-associated CpG islands is a well-documented epigenetic modification, acting as a mechanism to regulate gene expression associated with the development of cancer [6], [7]. Aberrant methylation of the tumor suppressor or DNA repair gene promoter has been detected in many different types of cancers [8], [9]. Methionine synthase, a vitamin B 12 -dependent enzyme, plays an important role in folate metabolism [10]. It catalyzes the remethylation of homocysteine to methionine and the concurrent demethylation of 5-methyltetrahydrofolate to tetrahydrofolate. Methionine synthase is critical for maintaining adequate intracellular methionine, an essential amino acid and the precursor of S-adenosylmethionine (SAM). SAM is a crucial methyl group donor involved in over 100 methylation reactions including DNA methylation. Recently, a polymorphism in the methionine synthase (MTR) gene (2756A→G, rs1805087), resulting in the substitution of aspartic acid (D919) by glycine (G), was identified in patients with methionine synthase deficiency and was found to be polymorphic among healthy controls [11]. In addition, Goode et al. suggested a modest inverse association between 2756GG polymorphism and homocysteine levels, indicating an increased enzymatic activity of the variant genotype [12]. Furthermore, a reduced homocysteine level was linked to the GG genotype in some studies [13]–[15], leading to the hypothesis that this polymorphism may have an activating effect on the enzyme that increases the conversion of homocysteine to methionine. Moreover, Paz et al. reported that individuals who carried 2756GG showed a lower frequency of CpG island hypermethylation in tumor suppressor genes [16].

Despite the biological plausibility of MTR functional polymorphism as a modulator of digestive system cancer susceptibility, previously inconsistent results have appeared in the literature. Published studies have generally been restricted in terms of sample size and ethnic diversity, and individual studies may have insufficient power to achieve a comprehensive and reliable conclusion. We therefore performed a meta-analysis of the published studies to clarify this inconsistency and to establish a comprehensive picture of the relationship between MTR and digestive system cancer.

## Materials and Methods

### Identification and Eligibility of Relevant Studies

Genetic association studies published before the end of Sep. 2012 on digestive system cancer and polymorphism within MTR gene were identified through a search of PubMed, EMBASE, ISI Web of Science, and CNKI (Chinese National Knowledge Infrastructure) without language restrictions using the following keywords and subject terms: ‘methionine synthase’ or ‘MTR’, ‘polymorphism’ or ‘variation’, and ‘cancer’ or ‘carcinoma’ or ‘neoplasm’. The titles and abstracts of potential articles were screened to determine their relevance, and any clearly irrelevant studies were excluded. The full texts of the remaining articles were read to determine whether they contained information on the topic of interest. Furthermore, reference lists of primary studies and review articles were also reviewed by a manual search to identify additional relevant publications. Studies included in the meta-analysis had to meet all the following criteria: (1) original papers containing independent data, (2) case–control or cohort studies, (3) association between MTR polymorphism and digestive system cancer risk was explored (4) identification of digestive system cancer cases was confirmed histologically or pathologically and (5) genotype distribution information or odds ratio (OR) with its 95% confidence interval (CI) and P-value. The major reasons for exclusion of studies were (1) overlapping data and (2) case-only studies, and review articles.

### Data Extraction

For each study, the following data were extracted independently by two authors: first author, year of publication, diagnosis criterion, age, sex, ethnicity, Hardy–Weinberg equilibrium (HWE) status, genotyping method, cancer type, source of control, total number of cases and controls, MTR polymorphism genotype counts and interactions between environmental factors or genes. The results were compared, and disagreements were discussed among all authors and resolved with consensus. If multiple published reports from the same study population were available, we included only the one with largest sample size and the most detailed information. Studies with different ethnic groups were considered as individual studies for our analyses.

### Statistical Analysis

Deviation from Hardy–Weinberg equilibrium was examined by Chi-square test with 1 degree of freedom. Crude Odds ratio (ORs) with corresponding 95% confidence intervals (CIs) were used to assess the strength of association between the MTR gene A2756G polymorphism and digestive system cancer risk. For the A2756G polymorphism, we investigated the association between genetic variants and digestive cancer risk in multiplicative model (G-allele vs. A-allele), dominant (AA+AG vs. GG) and recessive genetic model (GG vs. AA+AG). Between-study heterogeneity was measured using standard Q-statistic test [17]. Random-effects and fixed-effect summary measures were calculated as inverse-variance weighted average of the log odds ratio [18]. The results of random-effects summary were reported in the text because it takes into account the variation between studies. The Z test was used to determine the significance of the pooled OR. Subgroup analysis was stratified by the study characteristic according to ethnicity (East Asian, Caucasian and other), study design (hospital-based vs population-based) sample size (≥500 or <500 cases), genotyping method (RFLP vs others) and cancer type (colorectal cancer, esophagus cancer, gastric cancer, pancreatic cancer and hepatocellular carcinoma), respectively. Furthermore, meta-regression analysis was performed to investigate seven potential sources of heterogeneity including ethnicity, sample size, source of controls, genotyping method, cancer type, sex distribution among cases and controls, mean age of cases and controls [19]. Publication bias was investigated by funnel plot. Funnel plot asymmetry was assessed by the method of Egger’s linear regression test [20]. Sensitivity analysis, which determines the influence of individual studies on the pooled estimate, was determined by sequentially removing each study and recalculating the pooled relative risk and 95% confidence interval. Statistical analyses were done with the Stata software version 10.0 (Stata Corporation, College Station, TX). The type I error rate was set at 0.05. All P-values were two-tailed.

## Results

### Characteristics of Studies

**Figure 1:**
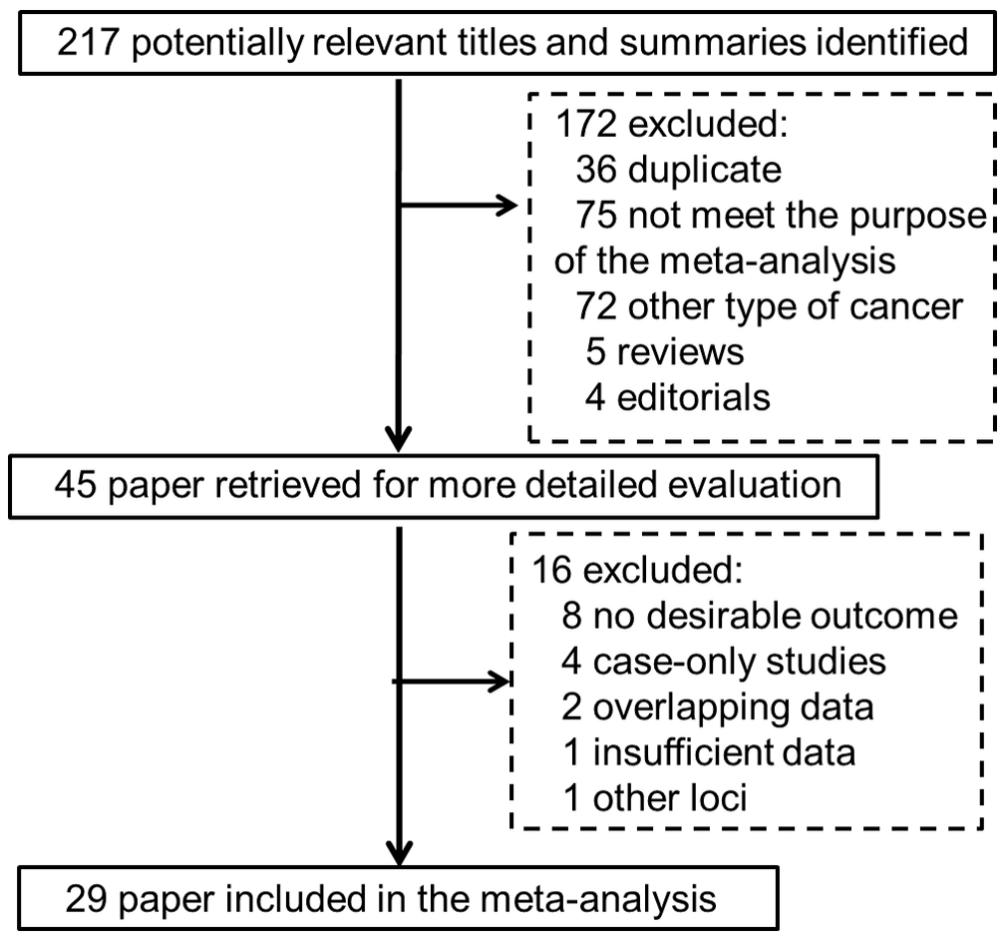
Flow diagram of the study selection process.

The combined search yielded 217 references. Study selection process was shown in Figure 1. Finally, a total of 29 studies with 34 data sets were finally included involving 15,368 patients and 23,959 controls [15], [21]–[48]. The detailed characteristics of the studies included in this meta-analysis are shown in Table 1. Of the cases, 82% were Caucasian, 16% were East Asian and 2% were other ethnic origins. The distribution of genotypes in the controls was consistent with Hardy–Weinberg equilibrium in all studies for MTR A2756G polymorphism.

**Table 1:** Characteristics of the studies included in the meta-analysis.

Study	Year	Ethnicity	Cancertype	Controlsource	No. ofcases/controls	Mean age ofcases/controls	Gender distributionin cases/controls(male %)	Genotypingmethod	P_HWE_ forcontrols
Ma [15]	1999	American	CRC	PB	356/476	NA/NA	100/100	RFLP	0.15
Le Marchand [21]	2002	American	CRC	PB	539/652	66.0/67.0	60.8/57.9	RFLP	0.54
Matsuo [22]	2002	Japanese	CRC	HB	142/241	NA/NA	58.9/49.0	RFLP	0.28
Pufulete [23]	2003	British	CRC	HB	28/76	68.9/58.0	46.0/45.0	RFLP	0.07
Ulvik [24]	2004	Norwegian	CRC	PB	2168/2192	NA/NA	63.5/63.5	Taqman	0.34
Matsuo [25]	2005	Japanese	CRC	HB	257/771	58.8/59.0	63.0/63.0	RFLP	0.4
Ulrich [26]	2005	American	CRC	PB	1600/1962	64.9/65.0	56.0/53.0	Taqman	0.13
Yang [27]	2005	Japanese	EC	HB	165/494	61.4/61.4	89.7/89.7	RFLP	0.43
Wang [28]	2006	Chinese	PC	HB	101/337	NA/NA	64.4/65.6	RFLP	0.86
Koushik [29]	2006	American	CRC	PB	363/804	68.2/68.0	47.6/42.0	Taqman	0.18
Chen [30]	2006	Chinese	CRC	PB	199/413	62.5/61.9	50.8/51.7	RFLP	0.18
Curtin [31]	2007	American	CRC	PB	916/1974	NA/NA	NA/NA	Taqman	0.09
Zhang [32]	2007	Polish	GC	PB	293/413	63.0/63.7	66.2/64.6	Taqman	0.22
Theodoratou [33]	2008	Scottish	CRC	PB	999/1010	62.3/62.7	57.3/56.9	Array	0.27
Guerreiro [34]	2008	Portuguese	CRC	PB	196/200	64.2/62.2	53.1/53.0	Taqman	0.41
Suzuki [35]	2008	Japanese	PC	HB	157/783	NA/NA	71.3/71.3	Taqman	0.56
Zhang [36]	2008	Chinese	CRC	HB	298/300	57.7/57.6	56.3/56.7	RFLP	0.13
Ott [37]	2008	German	EC, GC	HB	588/245	59.7/39	70.0/76.7	RFLP	0.97
Steck [38]	2008	American	CRC	PB	546/855	63.8/65.9	NA/NA	Taqman	0.14
de Vogel [39]	2009	Dutch	CRC	PB	696/1805	NA/NA	55.0/50.2	SNaPShot	0.31
Zhang [40]	2009	Chinese	CRC	HB	476/835	54.3/52.0	57.1/55.1	RFLP	0.67
Eussen [41]	2010	European	CRC	PB	1329/2364	58.9/58.7	51.0/53.0	Mass spectrometry	0.52
Levine [42]	2010	American	CRC	PB	1806/2879	53.5/54.0	51.3/44.4	iPLEX	0.17
Eussen [43]	2010	European	GC	PB	243/616	58.9/58.7	41.0/41.0	Mass spectrometry	0.12
Jokić [44]	2011	Croatian	CRC	PB	300/300	62.2/61.4	54.0/50.6	Taqman	0.82
Guimarães [45]	2011	Brazilian	CRC	PB	113/188	59.0/54.0	53.1/64.4	RFLP	0.06
Kim [46]	2011	Korean	CRC	HB	67/53	61.8/58.7	52.2/43.4	RFLP	0.12
Cui [47]	2012	Chinese	HCC	PB	356/641	56.6/58.7	83.1/43.5	RFLP	0.92
Martinelli [48]	2012	Italian	CRC	PB	71/80	69.0/58.0	59.2/53.8	RFLP	0.21

NA: not available, HB: hospital-based, PB: population-based, CRC: colorectal cancer, EC: esophagus cancer, HCC: hepatocellular carcinoma, GC: gastric cancer, PC: pancreatic cancer.

### Quantitative Data Synthesis

**Figure 2:**
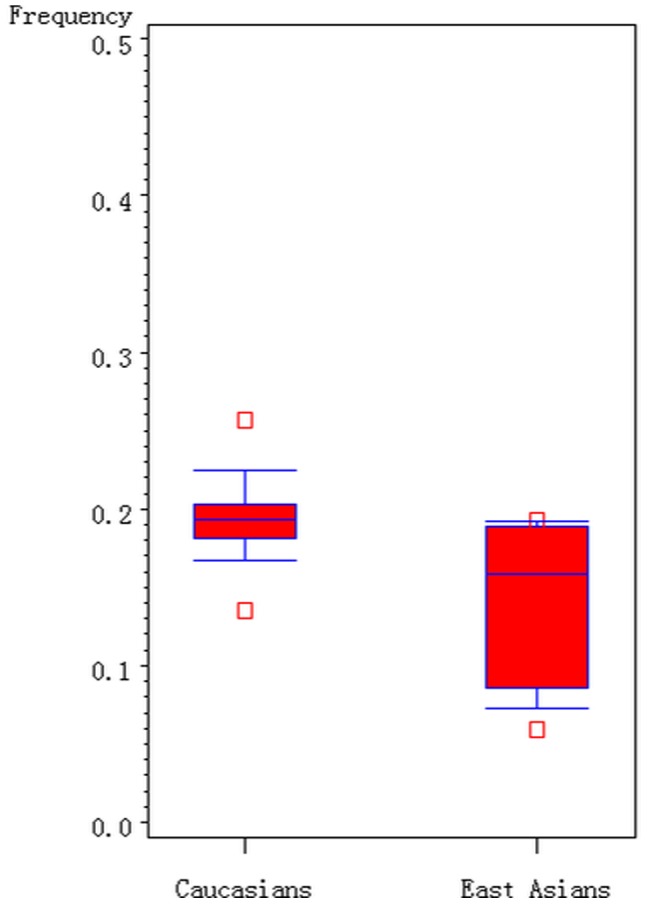
Frequencies of the G allele of MTR A2756G polymorphism among controls stratified by ethnicity.

As shown in Figure 2, the G allele distribution of the A2756G polymorphism varies among the controls across different ethnicities, ranging from 0.06 to 0.25. For East Asian controls, the G allele frequency was 0.14 (95% CI: 0.11–0.18), which was lower than that in Caucasian controls (0.20; 95% CI: 0.18–0.22), indicating a significant difference among East Asians as compared with Caucasians (P = 0.003).

Overall, there was no evidence of an association between the increased risk of digestive system cancer and the A2756G polymorphism in different genetic models when all eligible studies were pooled into the meta-analysis. Under random effect model, the per-allele overall OR of the G variant for digestive system cancer was 1.03 [95% CI: 0.98–1.09, *P*(Z) = 0.25, *P*(Q) = 0.05], with corresponding results under dominant and recessive genetic models of 1.03 [95% CI: 0.97–1.10, *P*(Z) = 0.33, *P*(Q) = 0.06] and 1.02 [95% CI: 0.89–1.17, *P*(Z) = 0.79, *P*(Q) = 0.25], respectively.

**Figure 3:**
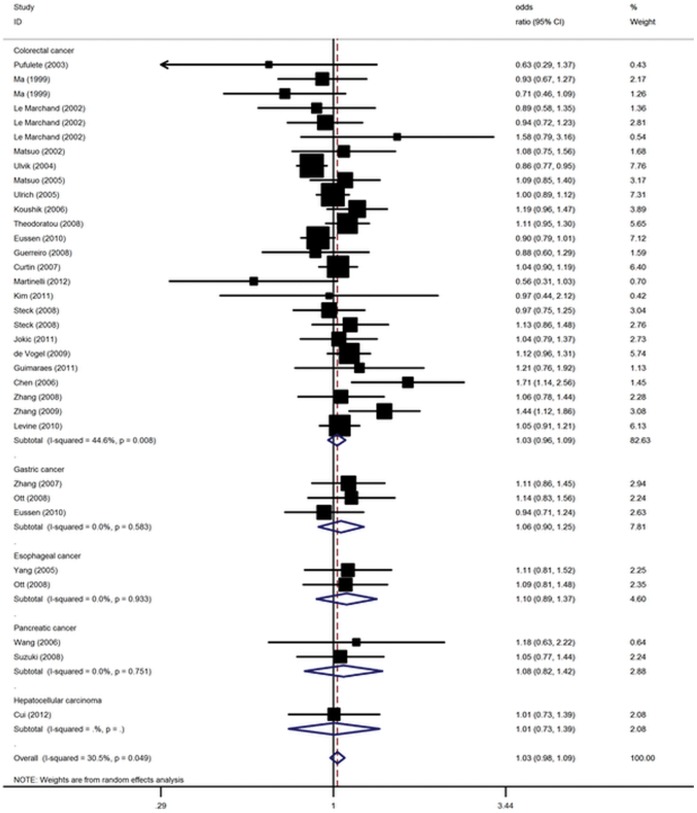
Forest plot from the meta-analysis of digestive system cancer and MTR A2756G polymorphism under random effect model.

This analysis is based on pooling of data from a number of different ethnic populations. When stratifying for ethnicity, an OR of 1.00 (95% CI: 0.94–1.05, P = 0.88) and 1.13 (95% CI: 1.02–1.25, P = 0.02) resulted for G allele, among Caucasians and East Asians, respectively. In the stratified analysis by cancer type, no significantly increased cancer risks were found for colorectal cancer, esophagus cancer, gastric cancer, pancreatic cancer and hepatocellular carcinoma in all genetic models (Figure 3). By considering control source subgroups, the OR was 1.01 (95% CI: 0.95–1.07, P = 0.78) in population-based controls, compared to 1.13 (95% CI: 1.02–1.25, P = 0.02) in hospital controls. In addition, no significant association between genotype of MTR A2756G and digestive system cancer risk in the stratified analysis according to sample size or genotyping method (Table 2).

**Table 2:** Main results of pooled odds ratios (ORs) with confidence interval (CI) in the meta-analysis.

Sub-group analysis	No. ofdatasets	No. ofcases/controls	G Allele			Dominant Model			Recessive Model		
			OR (95% CI)	P(Z)	P(Q)	OR (95% CI)	P(Z)	P(Q)	OR (95% CI)	P(Z)	P(Q)
Overall	34	15368/23959	1.03 (0.98–1.09)	0.25	0.05	1.03 (0.97–1.10)	0.33	0.06	1.02 (0.89–1.17)	0.79	0.25
Cancer type											
Colorectal cancer (Overall)	26	13465/20430	1.03 (0.96–1.09)	0.25	0.008	1.03 (0.96–1.12)	0.39	0.01	0.99 (0.85–1.15)	0.86	0.23
Colorectal cancer (Caucasians )	17	11396/17014	0.98 (0.91–1.05)	0.56	0.05	0.98 (0.91–1.06)	0.68	0.11	0.93 (0.91–1.06)	0.45	0.12
Colorectal cancer (Asians)	7	1754/3007	1.17 (1.00–1.36)	0.06	0.14	1.19 (0.98–1.45)	0.08	0.08	1.21 (0.84–1.77)	0.31	0.75
Gastric cancer	3	806/1029	1.06 (0.90–1.25)	0.50	0.58	1.04 (0.86–1.25)	0.72	0.42	1.30 (0.81–2.07)	0.27	0.73
Esophagus cancer	2	483/739	1.10 (0.89–1.37)	0.38	0.93	1.15 (0.89–1.48)	0.29	0.90	0.97 (0.48–1.93)	0.92	0.52
Pancreatic cancer	2	258/1120	1.08 (0.82–1.42)	0.60	0.75	1.01 (0.73–1.40)	0.95	0.81	2.80 (0.40–19.62)	0.30	0.10
Hepatocellular carcinoma	1	356/641	1.01 (0.73–1.39)	0.97	NA	0.96 (0.68–1.37)	0.83	NA	1.81 (0.52–6.30)	0.35	NA
Ethnicity											
Caucasian	21	12520/18288	1.00 (0.94–1.05)	0.88	0.10	0.99 (0.93–1.06)	0.87	0.16	0.96 (0.82–1.13)	0.62	0.18
East Asian	11	2533/5262	1.13 (1.02–1.25)	0.02	0.39	1.13 (0.99–1.29)	0.06	0.22	1.31 (0.96–1.79)	0.09	0.72
Other	2	315/409	1.18 (0.92–1.52)	0.20	0.37	1.18 (0.87–1.60)	0.30	0.39	1.53 (0.76–3.05)	0.23	0.72
Sample size											
<500	27	5854/9773	1.07 (1.00–1.14)	0.05	0.34	1.07 (0.99–1.16)	0.08	0.34	1.14 (0.95–1.38)	0.17	0.87
≥500	7	9514/14186	0.99 (0.92–1.08)	0.90	0.02	0.99 (0.90–1.09)	0.84	0.03	0.95 (0.73–1.25)	0.74	0.008
Source of control											
Population	23	13089/19824	1.01 (0.95–1.07)	0.78	0.03	1.01 (0.94–1.08)	0.83	0.04	0.98 (0.85–1.14)	0.83	0.24
Hospital	11	2279/4135	1.13 (1.02–1.25)	0.02	0.77	1.14 (1.01–1.28)	0.03	0.73	1.28 (0.93–1.75)	0.13	0.54
Genotyping method											
RFLP	19	3756/5802	1.07 (0.97–1.18)	0.13	0.20	1.08 (0.97–1.21)	0.16	0.18	1.08 (0.83–1.40)	0.57	0.59
Others	15	11612/18157	1.01 (0.95–1.07)	0.12	0.77	1.00 (0.94–1.07)	0.94	0.15	1.03 (0.86–1.23)	0.76	0.09

Although the formal test for heterogeneity was not significant in the overall analysis, we conducted meta-regression as there were also grounds for considering the ethnicity, sample size, genotyping method, cancer type, and clinical characteristics of cases and controls (age, and sex distribution) as potential sources of heterogeneity. In meta-regression analysis, ethnicity (P = 0.19), cancer type (P = 0.96), source of controls (P = 0.07), mean age of cases (P = 0.62) and controls (P = 0.72), genotyping method (P = 0.29) and gender distribution among cases (P = 0.65) and controls (P = 0.97) were not significantly correlated with the magnitude of the genetic effect. By contrast, the sample size (P = 0.008) was significantly correlated with between-study heterogeneity.

### Sensitivity Analyses and Publication Bias

In order to assess the stability of the results of the meta-analysis, we performed a sensitivity analysis through sequentially excluded individual studies. Statistically similar results were obtained after sequentially excluding each study, suggesting stability of the meta-analyses. Begg’s funnel plot and Egger’s test were performed to access the publication bias of the literatures. The shape of the funnel plots was symmetrical for the polymorphism (Figure S1). The statistic results also indicated a lack of publication bias of the current meta-analysis (Egger’s test: *P* = 0.25).

## Discussion

Large sample and unbiased epidemiological studies of predisposition genes polymorphisms could provide insight into the in vivo relationship between candidate genes and diseases. Methionine synthesis is the first step in the synthesis of SAM which is a universal methyl-group donor involved in methylation reactions including DNA methylation. This report is the first meta-analysis examining the effect of MTR A2756G polymorphism on the risk of digestive system cancer. In total, the meta-analysis involved 29 studies for digestive system cancer which provided 15,368 cases and 23,959 controls. Our results demonstrated that the G allele of the A2756G polymorphism on MTR is not a risk factor for developing digestive system cancer. Sensitivity analysis indicated robustness of our results.

In meta-analysis, heterogeneity evaluation was always conducted. Thus, subgroup meta-analyses were performed. In cancer type subgroups, no statistically significant association between MTR polymorphism and different types of cancer were found. However, in our meta-analysis, only one or two studies were available for some specific cancers, and they had limited sample size, and hence the results may be capricious and should be interpreted with caution. Meta-analysis is often dominated by a few large studies, which markedly reduces the evidence from smaller studies. However, in the stratified analysis according to sample size, no significant association between digestive system cancer susceptibility an MTR were found both in large and small studies for all genetic models. Besides, studies using different genotyping method also get consistent negative results.

In the stratified analysis by ethnicity, no significant associations were found in Caucasians for the polymorphism in all genetic models. However, we observed a marginal significant association between A2756G polymorphism and increased risk for digestive system cancer in East Asian populations. There are several explanations of this phenomenon. First, cancer is a complex disease and different genetic backgrounds may cause the discrepancy since the G allele distributions of the A2756G polymorphism varies between East Asian and Caucasian. In addition, different populations usually have different linkage disequilibrium patterns. A polymorphism may be in close linkage with another nearby causal variant in one ethnic population but not in another. MTR A2756G polymorphism may be in close linkage with different nearby causal variants in different populations. Moreover, clinical heterogeneity like age, sex ratio, dietary, years from onset and disease severity may also explain the discrepancy. Different populations may have differences in dietary intake of nutrients, some of which take part in the tumor formation.

Our results indicated that marginal significantly increased digestive system cancer risk in G allele carriers were found among the hospital-based studies but not in population-based studies. This reason may be that the hospital-based studies have some biases because such controls may just represent a sample of ill-defined reference population, and may not be representative of the general population very well, particularly when the genotypes under investigation were associated with the disease conditions that the hospital-based controls may have. Therefore, using a proper and representative population-based control subjects is very important to reduce biases in such genetic association studies.

Digestive system cancer is an extremely complex disease and the same polymorphism may have different roles in different tumor sites. Therefore, more studies for a specific type of digestive system cancer are needed to identify potential tumor-specific effect of MTR polymorphism. In addition, it is possible that the effect of a single polymorphism on digestive system cancer risk may be very small. Several other polymorphisms were identified, suggesting that possible combined effects of these polymorphisms on MTR activity may exist [49]. Furthermore, the effect of single genetic factor on the risk of digestive system cancer may be more pronounced in the presence of other common genetic or environmental risk factors such as alcohol abuse, smoking, hepatitis virus infection.

Compared with the previous meta-analysis [50], [51], the present study is much larger, with more than twice as many digestive system cancer cases as the earlier meta-analysis. In addition, several subgroup analysis and meta-regression analysis were conducted to identify potential source of heterogeneity.

Some limitations of this meta-analysis should be acknowledged. Firstly, the subgroup meta-analyses considering interactions between MTR genotype and different tumor type are based on a small number of studies with such information available. Secondly, our results were based on unadjusted estimates, while a more precise analysis should be conducted if individual-level data were available, which would allow for the adjustment by other covariates including drinking status, cigarette consumption, folate and vitamin B12 intake, family history, environmental factors and lifestyle [52]. Thirdly, only published studies were included in this meta-analysis. Therefore, publication bias may have occurred, even though the use of a statistical test did not show it. In spite of these, our present meta-analysis also had some advantages. First, no significant between studies heterogeneity were detected in all comparison. Second, no publication biases were found, indicating that the whole pooled results may be unbiased.

To conclude, our meta-analysis did not support an association of the A2756G polymorphism of MTR with digestive system cancer. For future association studies, well-designed studies with large sample size in diverse ethnic populations, more types of digestive system cancers along with tissue-specific biochemical, functional and expressional characteristics are required.

## Supporting Information

Figure S1 Begg’s funnel plot of MTR A2756G polymorphism and digestive system cancer.
(TIF)

Checklist S1 (DOC)
