# Supplementary figures and images for: Lack of Association between Methionine Synthase A2756G Polymorphism and Digestive System Cancer Risk: Evidence from 39327 Subjects
Source: PLoS One. 2013 Apr 16;8(4):e61511. doi: 10.1371/journal.pone.0061511 (PMC3629058; doi:10.1371/journal.pone.0061511)

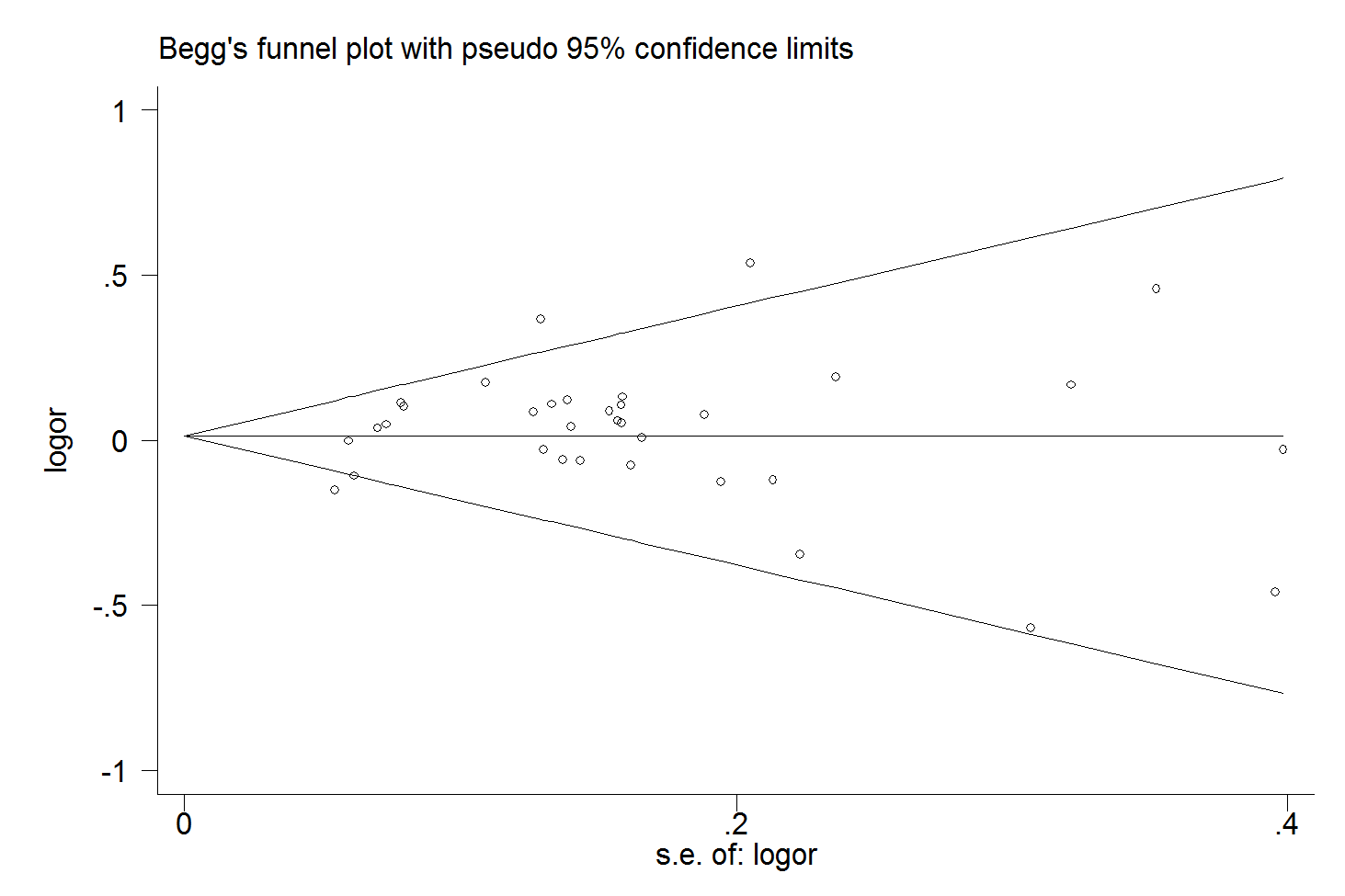

Supplement: Figure S1 — Begg’s funnel plot of MTR A2756G polymorphism and digestive system cancer. (TIF) [file pone.0061511.s001.tif]
